# A Study of the Fruits of *Catalpa bignonioides* Walt.: Evaluation of the Antioxidant, Anti-Inflammatory, and Anti-Cancer Activities in Colorectal Adenocarcinoma Cells in Relation to Phytochemical Profile

**DOI:** 10.3390/antiox14091116

**Published:** 2025-09-14

**Authors:** Clizia Bernardi, Thomas Gaslonde, Federica Finetti, Salim Benmaouche, Giulia Macrì, Annabelle Dugay, Claire Cuyamendous, Chouaha Bouzidi, Monica Rosa Loizzo, Philippe Belmont, Rosa Tundis, Lorenza Trabalzini, Brigitte Deguin

**Affiliations:** 1 Department of Biotechnology, Chemistry and Pharmacy, University of Siena, 53100 Siena, Italy; clizia.bernardi2@unisi.it (C.B.); giulia.macri@vismederi.com (G.M.); federica.finetti@unisi.it (F.F.); lorenza.trabalzini@unisi.it (L.T.); 2 Faculté de Pharmacie de Paris, Université Paris Cité, U.M.R. CiTCoM (n°8038—CNRS/Université Paris Cité), F-75006 Paris, France; thomas.gaslonde@u-paris.fr (T.G.); salim.benmaouche@u-paris.fr (S.B.); annabelle.dugay@u-paris.fr (A.D.); claire.cuyamendous@u-paris.fr (C.C.); chouaha.bouzidi@u-paris.fr (C.B.); philippe.belmont@u-paris.fr (P.B.); 3 Department of Pharmacy, Health and Nutritional Sciences, University of Calabria, 87036 Rende, Italy; monica_rosa.loizzo@unical.it (M.R.L.); rosa.tundis@unical.it (R.T.)

**Keywords:** iridoids, chemical profile, HPLC-DAD-MS, HPTLC bioautography, antioxidants, HT29 cells

## Abstract

The chemical profiles and potential anti-inflammatory, antioxidant, and anticancer activities of the aqueous extract and fractions of fresh *Catalpa bignonioides* fruits were studied. Iridoids, flavonoids, and phenolic compounds represent the main phytochemical classes. Nine of the ten iridoids detected are acyl-iridoids. Significant amounts of catalpol and catalposide were found. The antioxidant activity of iridoids was demonstrated by HPTLC analysis coupled with a DPPH derivatization and by applying four in vitro tests, such as DPPH, ABTS, FRAP, and the β-carotene bleaching test. *C. bignonioides* extract and fractions were also evaluated for their anti-cancer activity using in vitro models of colorectal cancer (HT29 and HCT166 cell lines), and focusing on the effect of the different fractions on inflammation and oxidative stress, key factors that drive the onset and progression of colon cancer.

## 1. Introduction

Iridoids, an important class of metabolites with a cyclopenta[*c*]pyran skeleton, are found in numerous botanical families, many of which include medicinal or food plants renowned for their role in healthcare. Plant extracts containing iridoids are well-known in traditional medicine for their bitter tonic, sedative, antitussive, hypotensive, antidiabetic, antipyretic, and anti-arthritic properties, as well as for treating wounds, skin disorders, and occasionally cancer.

Numerous studies have identified iridoids as the primary bioactive compounds in these extracts, exhibiting potent pharmacological and biological activities. These compounds are considered a promising source of anti-inflammatory, antioxidant, neuroprotective, cardioprotective, and anti-cancer agents, often characterized by low toxicity [1].

Since inflammation is associated with the development of cancer in humans, several studies have highlighted the potential of iridoids in combating various types of cancer, including colorectal cancer. These studies have demonstrated the efficacy of iridoids in cancer treatment and have explored the molecular mechanisms underlying their pharmacological activity [2,3].

Among the multitude of iridoids described in the literature, we focused on benzoyl and cinnamoyl acyl-iridoid glycosides bearing phenol groups, which have revealed interesting radical-scavenging, anti-inflammatory, and anti-cancer activities. These compounds, including acyl-catalpol derivatives, are produced by plants used in traditional medicine for these properties, belonging to the genera *Harpagophytum*, *Verbascum*, *Picrorhiza*, *Catalpa*, *Tabebuia*, and *Veronica* [4,5]. The examination of the chemotaxonomic distribution of iridoids in the plant kingdom shows that these bioactive terpenoids are not only limited to medicinal plants but are also produced by plants grown for ornamental purposes.

Phytochemical studies on species belonging to the *Catalpa* Scop. genus (Bignoniaceae family), have revealed the richness of catalpol derivatives in polar extracts [6]. Among the species belonging to this genus, *Catalpa bignonioides* Walt., also known as cigar tree, bean tree, or Indian catalpa, is widely cultivated worldwide. Native to the southeastern United States, it has been introduced to other continents. This medium-sized deciduous tree with a height of 15–18 m is grown as an ornamental tree along streets and in gardens in many temperate regions worldwide.

According to the descriptions of its properties and traditional uses listed in Cook’s Physiomedical Dispensary (1869) [7], this tree appears to have been important in the medical practice of indigenous cultures. More recently, various studies carried out on extracts have highlighted promising anti-inflammatory, antinociceptive [8] and antioxidant [9] activities and a potential effect on muscle growth and strength improvement [10]. The potential use in the management of diabetes by contributing to glucose homeostasis [11] was also demonstrated.

It has been shown that the main biological effects of *Catalpa* extracts are attributable to iridoid glycosides [12,13,14]. However, several studies on certain species belonging to the *Catalpa genus* [15], including *C. speciosa*, *C. ovata*, and *C. bungei*, have revealed that other classes of metabolites may contribute to biological activities, including anti-cancer activity, as demonstrated for phenols [16] and naphthoquinones [17,18,19]. While previous studies have highlighted similarities in the phytochemical composition and biological activities across the different *Catalpa* species, the study of *C. bignonioides* fruits is still limited.

To our knowledge, their anti-cancer activity has not yet been explored. In this context, this work investigates the chemical profile of *C. bignonioides* fruits and evaluates their potential anti-inflammatory, antioxidant, and anti-cancer activities.

## 2. Materials and Methods

### 2.1. Chemicals and Reagents

Liquid chromatography-mass spectrometry (LC-MS) quality grade methanol, acetic acid, sulfuric acid, ethyl acetate, formic acid, isopropanol, *n*-butanol, toluene, dichloromethane, and acetonitrile were purchased from Carlo Erba (Val de Reuil, France). Standard compounds, such as quercetin (purity > 99%), rutin (purity > 99%), minecoside (purity > 90%), catalpol (purity > 90%), and picroside III (purity > 98%) were purchased from Extrasynthese (Genay, France). Pure catalposide, catalpol, minecoside, and specioside were isolated from the fruits of *C. bignonioides*. Vanillin and magnesium chloride hexahydrate (MgCl_2_), 2,2-diphenyl-1-picrylhydrazyl (DPPH), 2,4,6-tripyridyl-*s*-triazine (TPTZ), 2,2′-azino-bis(3-ethylbenzothiazoline-6-sulfonic acid) diammonium salt (ABTS) solution, potassium persulfate, acetate buffer, β-carotene, ascorbic acid, butylated hydroxytoluene (BHT), and 2-aminoethyl diphenylborinate (NP) were purchased from Sigma Aldrich, a subsidiary of Merck KGaA (Darmstadt, Germany); polyethylene glycol 4000 (PEG) was purchased from Ferak (Berlin, Germany). RPMI-1640 and DMEM media, Fetal Bovine Serum (FBS), penicillin, streptomycin, L-glutamine, L-glucose, PBS, and TBS buffers, the BCA protein assay kit, and the ECL detection kit were purchased from Euroclone (Pero, MI, Italy). Trypan Blue was from Twin Helix (Rho, MI, Italy). Interleukin 1β (IL1β) was purchased from Vinci Biochem (Vinci, FI, Italy). 3-(4,5-dimethylthiazol-2-yl)-2,5-diphenyltetrazolium bromide (MTT), Dimethyl sulfoxide (DMSO), and Tween-20 were purchased from Sigma-Aldrich (St. Louis, MO, USA). The Laemmli Sample Buffer was purchased from Bio-Rad (Hercules, CA, USA). Radioimmunoprecipitation assay (RIPA) buffer, primary antibodies, and horseradish peroxidase-conjugated secondary antibodies were purchased from Cell Signaling Technology (Danvers, MA, USA). 2,7- Dichlorodihydrofluorescein diacetate (DCFH2-DA) was from ThermoFisher (Waltham, MA, USA).

### 2.2. Plant Materials

Fruits of *C. bignonioides* were collected in the Botanical Garden of Université Paris Cité, Faculté de Pharmacie de Paris (Latitude: 48°51′24″ N, Longitude: 2°21′07″ E, referenced R71 in Botanical Garden Register) in September 2017 and at the end of August 2023.

Fruits were randomly selected and examined for integrity and absence of dust and insect contamination. Samples were harvested at the maturity stage, defined by visual color and size measurement. The fresh fruits were weighed and then stored frozen.

### 2.3. Extraction and Fractionation

Fresh frozen fruits of *C. bignonioides* (1.0 kg) were gradually immersed in 4 L of boiling water to maintain boiling. After two hours, the extract was filtered, and the pomace was reprocessed 3 times with 2 L of water under the same conditions. The aqueous extracts were combined and concentrated under vacuum to a final volume of 1 L. The concentrated aqueous phase (EAC) was then extracted with *n*-butanol (1 L), and the operation was repeated 3 times with 0.5 L of *n*-butanol. The residual aqueous phase (AP) was separated and stored. The *n*-butanolic extracts were combined and evaporated to obtain a pourable syrup (EBC). One liter of dichloromethane was vigorously stirred, and the resulting EBC syrup poured in. A yellowish solid began to precipitate. After 30 min, the solid was filtered and washed several times with dichloromethane. As the precipitate was very hygroscopic, it was dissolved in 200 mL of water and lyophilized to give a light, crisp yellowish powder (EDC) (yield 2.5% from fresh material). EAC, EBC, and EDC samples were dried and kept frozen.

The residual aqueous phase (AP) was concentrated and then freeze-dried. The very hygroscopic brown crude (100 g) was dissolved in methanol, and the insoluble part was filtered. 200 g of high particle-size silica (0.063–0.200 mm) was added, and the suspension was evaporated to prepare a solid deposit. After vacuum liquid chromatography (VLC) (height = 15 cm; ⌀ = 25 cm; silica (0.015–0.04 mm); CH_2_Cl_2_/MeOH 9/1 (*v*/*v*)) [20], the catalpol-containing fractions of acceptable purity by TLC analysis were combined and evaporated under reduced pressure. A slightly yellowish powder (14 g, 1.4% from fresh material) was obtained. During VLC, a pure fraction of catalpol was collected and stored separately.

These two steps were repeated on the 2023 harvested batch only to confirm HPLC profiles and quantities of major iridoids. All other analyses were carried out on samples obtained from the 2017 harvest of frozen fruit.

A preparative centrifugal partition chromatography (CPC) performed on an SCPC-250+1000-B, equipped with a one-liter rotor containing 2016 twin-cells (Armen Instrument, Saint-Avé, France), was carried out using a solvent system (ethyl acetate/acetonitrile/water 2/1/3 (*v*/*v*/*v*)) in ascending mode. The CPC column was initially filled with the aqueous stationary phase at 1200 rpm. The dichloromethane extract (4.0 g) dissolved in a mixture of 10 mL of the aqueous layer and 10 mL of the organic layer was injected. After elution with the organic layer at 12 mL/min, followed by extrusion of the stationary phase with an aqueous layer, fractions were collected according to their TLC profiles. Fraction A (222 mg) contained mainly *p*-hydroxybenzoic acid. Fraction B (115 mg), fraction C (228 mg), and fraction D (2.05 g) contained three different iridoids. After purification with additional preparative HPLC (Macherey Nagel Nucleodur C18 HTec, 250 × 32 mm, 5 μm, MeOH/H_2_O at 35 mL/min), samples with analytical purities were obtained. NMR spectra were in accordance with literature data and permitted to assign the formula of minecoside to fraction B, specioside to fraction C, and catalposide to fraction D.

### 2.4. High Performance Liquid Chromatography (HPLC) Analyses

Dry EAC, EBC, and EDC samples of *C. bignonioides* were dissolved in methanol before being analyzed by HPLC-DAD-MS and HPLC (U-3000, Thermo, Courtaboeuf, France) coupled to an ESI-QTOF-MS (Maxis II, Bruker, Champs sur Marne, France) (EAC: 51 mg/5 mL; EBC: 25 mg/5 mL and EDC: 12 mg/5 mL) and filtered through Econo filter Nylon 13 mm 0.2 µm (Agilent, Les Ulis, France). Qualitative analysis of *C. bignonioides* samples was performed using an HPLC (U-3000, Thermo, Courtaboeuf, France) coupled to an ESI-QTOF-MS (Maxis II, Bruker, Champs sur Marne, France) on a C18 column (Acclaim RSLC polar advantage II, 100 × 2.1 mm, 2.2 μm) at 35 °C.

The mobile phase was a mixture of 0.1% formic acid, 10% methanol and water (phase A), and 0.1% formic acid and acetonitrile (phase B). The elution gradient was: 0 to 2 min 95% A; 2 to 7 min, 95 to 85% A; 7 to 15 min, from 85 to 50% A; 15 to 18 min, 50 to 20% A; 18 to 19 min, 20% and 19 to 21 min, 20 to 95% A. The injection volume was 2 μL, and the flow rate was 0.3 mL/min. Chromatograms were obtained at 240, 270, 340, and 510 nm. Mass spectra were acquired in positive mode by using the following parameters: ESI 3500 V, m/z 50–1200, MS 2 Hz.

Qualitative analysis of *C. bignonioides* samples was also performed using an HPLC-DAD-MS Thermo Scientific Dionex U3000 (Thermo-Dionex, Les Ulis, France) including a quaternary pump (LPG-3400 SD), an autosampler thermostat (WPS-3000TSL), a column thermostat (TCC-3000SD), and a Diode Array Detector (DAD-3000) (Thermo-Dionex, Les Ulis, France), on line with a quadrupole mass spectrometer (Surveyor MSQ plus System, Thermo-Dionex, Les Ulis, France).

The analytical column was a C18 Acclaim Polar Advantage II (Thermo Scientific, Courtaboeuf, France), 100 × 2.1 mm, 3 μm thermostated at 35 °C during the analysis.

Two chromatographic mobile phases were employed for a gradient elution as follows: solvent A: 0.1% formic acid in ultrapure water containing 10% LC-MS grade methanol (*v*/*v*), solvent B: 0.1% formic acid in acetonitrile LC-MS grade (*v*/*v*). In the mobile phase, the gradient was applied as follows: 95:5 from 0 to 45 min, 80:20 between 45 and 50 min, then 20:80 between 50 and 60 min, and finally, between 60 and 65 min, return to initial conditions. The pump flow rate was set at 0.6 mL/min; the sample injection volume was 10 µL. Detection at specific wavelengths 210, 254, 280, and 350 nm was used to record the chromatograms.

The chromatographic effluent carried by a stream of nitrogen was directed into the electrospray ionization source of the mass spectrometer (MS). The MS was operated in the positive and negative ionization modes with the following operating conditions: ion spray voltage 3 kV, curtain gas 50 psi, Q energy was 70 V, cone voltage 50 V, desolvation temperature 500 °C, and ion energy 0.8 V. Chromeleon version 6.8 software, provided by Thermo Scientific Dionex, (Les Ulis, France) was used for obtaining results.

### 2.5. Quantification of Catalposide

The quantification of catalposide in the EAC, EBC, and EDC samples was carried out under the chromatographic conditions described above, using a Thermo Scientific Dionex U3000 HPLC-DAD-MS instrument. The quantification method used was internal calibration at 260 nm, which is the maximum wavelength of the UV spectrum of catalposide. Initially, a calibration curve (Abs = f(C)) was constructed with six different concentrations of pure catalposide (pC), isolated and characterized from *C. bignonioides* fruits. At the six different concentrations prepared in water of pure catalposide C0 = 0.950 g/L; C1 = 0.8 g/L; C2 = 0.5 g/L; C3 = 0.4 g/L; C4 = 0.2 g/L; C5 = 0.04 g/L, an internal standard was added, i.e., a quantity of commercial quercetin at a final and constant concentration of 1.0 g/L.

The regression equation and a correlation coefficient r^2^ were obtained from the ratio of the areas of the peaks of the standard catalposide compound and the internal standard quercetin compound, respectively. Then, a HPLC analysis was performed, keeping the same conditions used previously. Quercetin concentration of 1.0 g/L was kept constant in all samples EAC, EBC, and EDC dissolved in water (CEAC = 10.2 g/L; CEBC = 5 g/L, and CEDC = 2.4 g/L). The analysis for each sample was repeated three times. Using the *ratio* of the areas of catalposide and quercetin present in the three extracts and the regression equation previously obtained, the concentration of catalposide in each of the three extracts was quantified. The linear regression analysis is as follows: Surface catalposide/Surface quercetin = 1.4468 × C (g/L) with a correlation coefficient r^2^ > 0.990. To validate the method, the limit of detection, LOD = 2.4 + 0.4 (mg/L), was calculated from 3× (signal-to-noise ratio). The limit of quantification, LOQ = 8 + 0.1 (mg/L), was calculated using 10× (signal-to-noise ratio). The repeatability of the range points was also assessed, with a coefficient of variation of less than 3.6% for concentrations ranging from 0.04 to 1.5 g/L. For calalposide samples, the intraassay precision of the unknown concentrations was less than 2.2%. This shows that the assay method is sensitive and robust.

### 2.6. High-Performance Thin-Layer Chromatography (HPTLC) Analysis

Each compound was weighed, dissolved in methanol, sonicated, and filtered through a 13 mm 0.2 µm Econo Nylon filter (Agilent, Les Ulis, France). HPTLC analyses were performed on 20 × 10 cm HPTLC glass plates silica gel 60 F254 (Merck KGaA) with an HPTLC system (CAMAG) controlled by vision CATS 3.0 software, and consisting of an automatic TLC sampler ATS 4, an automatic developing chamber ADC 2, a TLC plate heater III, a TLC derivatizer, and a TLC visualizer 2.

Each sample (5.0 µL) was applied at a rate of 150 nL/s as a 5 mm band, 8 mm apart and 8 mm from the lower edge, resulting in 9 tracks, with the first one at 28 mm from the left edge of the plate. After conditioning for 10 min at 33% relative humidity (saturated MgCl_2_ solution), the plates were developed in a saturated chamber (20 min, with filter paper) to a distance of 70 mm from the lower edge of the plate, using ethyl acetate-methanol-water (77/15/8, *v*/*v*/*v*) mobile phase. The developed plates were dried for 5 min with a stream of air.

For derivatization with vanillin, 2 mL of vanillin sulfuric acid reagent was sprayed using level 4 of the yellow nozzle. Derivatized plates were heated (CAMAG TLC Plate Heater III) at 100 °C for 3 min until color developed. For derivatization with NP-PEG reagent, 2 mL of NP reagent and 2 mL of PEG reagent were sprayed consecutively, using level 4 of the green nozzle and level 3 of the blue nozzle, respectively.

The developed plates were heated (CAMAG TLC Plate Heater III) at 100 °C for 3 min, then 4 mL of DPPH reagent 0.05% solution in methanol (50 mg of DPPH reagent was added in 100 mL of methanol) was manually sprayed. Plates after the derivatization process were dried in the dark with aluminum foil for 5 min. Digital images of the plates were recorded in ultraviolet (UV) 254 nm (short-wave UV), UV 366 nm (long-wave UV), and white light after development and after derivatization.

### 2.7. Cell Culture

Colorectal adenocarcinoma cells HT29 (ATCC, Rockville, MD, USA) were cultured in RPMI-1640 medium supplemented with 10% FBS, 100 U/mL penicillin/streptomycin, and 4 mM L-glutamine. HCT116, colorectal carcinoma cells (ATCC, Rockville, MD, USA) were cultured in DMEM with 4500 mg/L glucose supplemented with 10% FBS, 100 U/mL penicillin/streptomycin, and 4 mM L-glutamine. All cell lines were grown at 37 °C and 5% CO_2_.

### 2.8. Trypan Blue Assay

HT29 cells (7.5 × 10^5^ cells/mL) were suspended in RPMI 0.1% FBS and treated with EAC, EBC, or EDC, at different concentrations (0.01–0.1–1–10–100 µg/mL). After 48 h, one part of the cell suspension was mixed with one part of 0.4% trypan blue in a microtube and counted using a Thoma chamber. The result was expressed as the number of dead cells to the total number of cells and are representative of three different experiments run in triplicate [21].

### 2.9. MTT Assay

Either 3.5 × 10^3^ (HT29) and 2.5 × 10^3^ (HCT116) cells/well were seeded in 96-multiwell plates in medium with 10% FBS, and after adherence, were maintained for 24 h in medium containing 0.1% FBS. After 24 h, cells were treated with EAC, EBC, or EDC (0.01, 0.1, 1, 10, 100 µg/mL) and with catalposide (0.01, 0.1, 1, 10 µg/mL) in the presence/absence of 10 ng/mL IL1β. After 48 h, the medium was removed and replaced with fresh medium in the presence of 1.2 mM MTT for 4 h. Subsequently, once the MTT solution was removed, the formazan salts were solubilized in 50 µL of DMSO. Cell viability was evaluated by measuring the absorbance at 595 nm using a microplate reader (EnVision, PerkinElmer, Waltham, MA, USA). Data were expressed as a percentage of the basal control and are representative of three different experiments run in triplicate [22].

### 2.10. Clonogenic Assay

HT-29 cells were plated in 6 multi-well plates at a concentration of 750 cells/well in medium containing 10% FBS. After 24 h, cells were pre-treated for 1 h with EAC, EBC, or EDC (10–100 µg/mL). Then, 10 ng/mL IL-1β was added, and cells were kept in incubator for 10 days. Colonies were fixed with methanol, stained, and counted (>50 cells). Data were expressed as a percentage of the basal control and are representative of three different experiments run in triplicate [22].

### 2.11. Western Blotting Analysis

HT29 cells (3.5 × 10^5^ cells/well) were seeded in a 60 mm dish in medium with 10% serum. After 24 h, cells were starved for 24 h in medium containing 0.1% serum, and then treated with EAC, EBC, or EDC (10–100 µg/mL), with or without IL1β (10 ng/mL). After 48 h, the extraction of total proteins was performed by lysing cells in precooled RIPA buffer. Cell lysates were centrifuged at 13,000× *g* for 15 min at 4 °C, and protein concentration was determined using the BCA method. Subsequently, equal amounts of proteins (50 µg) were treated with Laemmli Sample Buffer, heated for 5 min, separated by 10% sodium dodecyl sulfate (SDS)-polyacrylamide gel electrophoresis (PAGE), and transferred into a nitrocellulose membrane using a Semidry Electro-blotter System (Galileo Bioscience, Cambridge, MA, USA).

Unspecific protein binding sites were blocked by incubation with 5% milk and 0.5% Tween-20 in TBS at room temperature for 1 h. Membranes were then incubated overnight at 4 °C with appropriate dilutions of anti-COX2 or anti-β-actin primary antibodies. Subsequently, membranes were incubated for 1 h with appropriate horseradish peroxidase-conjugated secondary antibodies. Immunoreactive proteins were then visualized by ECL detection system, and images were digitized with Image Quant LAS4000 (GE Healthcare Europe GmbH, Milano, Italy). Immunoreactive bands from Western blots were quantified by densitometry using ImageJ 1.52a Java 1.8.0_112 (64-bit) (open-source image processing program, National Institutes of Health, Bethesda, MD, USA) and are representative of five different experiments [23].

### 2.12. ROS Measurement

5.0 × 10^4^ cells/well (HT29) were seeded in 24-multiwell plates and, after adherence, were pre-treated for 1 h in medium containing 0.1% FBS with EAC, EBC, or EDC (100 µg/mL) and then exposed to IL1β (10 ng/mL). After 24 h, cells were trypsinized and suspended with 10 µM DCFH2-DA for 15 min. Cells were spun and resuspended in PBS. The suspension was added in a 96-multiwell plate, and intracellular levels of ROS were evaluated photometrically with a CLARIOstar microplate reader (BMG LABTECH) (excitation/emission 495/527). Data are representative of three different experiments run in triplicate

### 2.13. In Vitro Antioxidant Properties

β-Carotene bleaching test, FRAP (Ferric Reducing Antioxidant Power) test, ABTS (2,2′-azino-bis (3-ethylbenzothiazoline-6-sulfonic acid) diammonium salt) test, and DPPH (2,2-diphenyl-1-picrylhydrazyl) test were applied to investigate the potential antioxidant effects of *C. bignonioides* extracts and catalposide [24].

#### 2.13.1. β-Carotene Bleaching Test

The β-carotene bleaching test was used to evaluate the ability of *C. bignonioides* extracts and catalposide to counteract lipid peroxidation. In this assay, 20 μL of linoleic acid and 200 μL of 100% Tween 20 were added to 1 mL of a β-carotene solution (0.2 mg/mL in chloroform). After evaporation of chloroform and dilution with water, 5 mL of the emulsion was transferred into different test tubes containing 200 μL of samples in 70% ethanol at concentrations in the range from 2.5 to 100 μg/mL. After incubation at 45 °C for 30 min, the absorbance was read at 470 nm. Results were expressed as sample concentrations able to scavenge 50% of DPPH radicals (IC_50_).

#### 2.13.2. FRAP Test

The FRAP test is based on the redox reaction that involves the TPTZ (2,4,6-tri(2-pyridyl)-*s*-triazine)-Fe^3+^ complex. FRAP reagent was mixed with a sample dissolved in ethanol at a concentration of 2.5 mg/mL. After half an hour of incubation at 25 °C, the absorbance was read at λ = 595 nm using a UV-Vis Jenway 6003 spectrophotometer (Carlo Erba, Milan, Italy). Results were expressed as μM Fe (II)/g.

#### 2.13.3. ABTS Test

2,2′-Azino-bis (3-ethylbenzothiazoline-6-sulfonic acid) diammonium salt radical cation (ABTS^·+^) is a stable free radical frequently used for estimating the total antioxidant capacity (TAC) of natural products. In the ABTS test, to obtain a solution of ABTS radical cation, ABTS solution (7 mM, 50 mL) and potassium persulfate (2.45 mM, 500 μL) were mixed and left to react overnight. After that, the solution was diluted with ethanol to reach a final absorbance of 0.70, measured at 734 nm. For the test, extracts and catalposide at different concentrations from 0.2 to 200 μg/mL (25 μL) were mixed with 2000 mL of diluted ABTS^+^ solution and left to react for 6 min at room temperature before measuring the absorbance at 734 nm. Results were expressed as sample concentrations able to scavenge 50% of DPPH radicals (IC_50_).

#### 2.13.4. DPPH Test

In the DPPH assay, samples (at concentrations in the range 5–250 μg/mL) were mixed with a DPPH solution (1.0 × 10^−4^ M). After half an hour at room temperature, the absorbance was measured at 517 nm (UV-Vis Jenway 6003 spectrophotometer, Carlo Erba, Milan, Italy). Results were expressed as sample concentrations able to scavenge 50% of DPPH radicals (IC_50_).

### 2.14. Statistical Analysis

Data from biological investigations were the result of different experiments and were expressed as mean ± standard deviation (SD). Statistical analysis was performed using Student’s t-test, One-way analysis of variance test (ANOVA), followed by a multicomparison Dunnett’s test or Tukey’s multiple comparisons test (GraphPad Prism 8.4.3) (San Diego, CA, USA). Any differences in the dataset of *p* < 0.05 were considered statistically significant [23]. The concentration giving 50% inhibition (IC_50_) was calculated by nonlinear regression using Prism GraphPad version 4.0 for Windows (San Diego, CA, USA).

## 3. Results and Discussion

*Catalpa bignonioides* is an ornamental tree well suited to the city and the garden, much appreciated for the shade it provides. Its dense foliage, adorned with broad deciduous leaves, puts on a floral show in July. It is adorned with clusters of white flowers striped with red and resembling trumpet-shaped bells. The catalpa is also renowned for its surprising fruiting: at the end of August, it forms long green fruits 30 to 40 cm long, which turn brown in autumn, earning it the name “bean tree”.

To obtain the results described in this work, it was necessary to harvest the fruit at the ripening stage, defined by a visual green color, because when the fruit starts to turn brown, the quality of the extracts is altered. The bioactive compounds are no longer produced, or their availability becomes very low. Genus *Catalpa* contains several classes of metabolites such as flavonoids, phenolic acids, phenols, triterpenes, fatty acids, iridoids, lignans, quinones (listed in REAXYS database), and tannins [25].

To ensure that extracts were rich in iridoid glycosides esterified with benzoic and cinnamic phenolic acids, extraction was carried out on fresh-frozen material using the Bourquelot and Herissey process [26] to prevent potential hydrolysis of iridoid heterosides. The compounds of interest were then isolated from the aqueous extract by liquid–liquid extraction using *n*-butanol and then precipitated in dichloromethane. A sample at each stage was taken and preserved for phytochemical and biological analysis; these samples were named EAC, EBC, and EDC, respectively.

### 3.1. Chemical Composition of C. bignonioides Fruit Extracts

Several phytochemical studies have revealed that *C. bignonioides* extracts contain flavonoids, iridoids, lignans, oligosaccharides, phenolic acids, phenolic glycosides, and phenylethanoid diglycosides. These compounds are described in roots [27], petioles and leaves [28,29,30], as well as in fruits [11]. In addition, naphtoquinone-type compounds, as catalponol derivatives, are known to be present in heartwood extracts [31].

To identify the main metabolites of the three *C. bignonioides* fruit samples (EAC, EBC, and EDC), two types of LC-MS analyses were carried out to correlate quasi-molecular ions, fragment ions, and their UV absorbances. Table 1 presents the results of the three samples obtained by HPLC coupled with ESI-QTOF-MS analysis. The software Chromeleon 6.8, provided by Thermo Scientific (91940 Les Ulis, France) was used for data treatment of ESI-QTOF-MS analysis.

Compound identification was established from quasi-molecular ions [M+H]^+^ and fragment ion values. From the exact experimental weight values, the molecular formula and its exact molecular weight were found and confirmed by error values < 10 ppm and precision value < 20 m Sigma. Table 2 shows the results of the HPLC-DAD-MS profile, i.e., the compounds identified based on UV spectra, quasi-molecular ions, and adducts ([M-H]^−^/[M+45]^−^). The results of both analyses are consistent and in agreement with the phytochemical composition of catalpa species. The aqueous extract (EAC) is the richest in identified metabolites. EAC analysis revealed the presence of flavonoids, iridoids, phenylpropanoid glycosides, and phenolic acids as the main constituents of the fruit.

Ten iridoids were detected in the EAC sample, nine of which are acyl-iridoids (Table 3). Of these, catalposide, verproside, verminoside, minecoside, specioside, and picroside III are acyl-catalposides.

All the iridoids identified in the EAC extract have already been described in the *Catalpa* genus. Eight of the ten iridoids have already been found in different organs of *C. bignonioides*. To our knowledge, this is the first time that the verproside and verminoside have been identified in an extract from this tree. However, these two iridoids were previously identified in a *C. ovata* [32] whose phytochemical composition has been studied in greater detail. Extraction with *n*-butanol (EBC sample) proved to be selective for acyl-iridoids, as the more polar catalpol was not found in the EBC and EDC samples. To confirm the nature of the iridoids and to obtain sufficient quantities of control samples useful for the various analyses in this study, we were able to isolate pure catalposide, minecoside and specioside from a batch of EDC using a series of separative techniques, and catalpol using a VLC procedure from the residual aqueous phase (NMR data, see Supplementary Materials**)**

Flavonoid glycosides have also been extracted using boiling water. The first studies describing the presence of flavonoids of the *Catalpa* genus were carried out by Birkofer et al. [33] and Harbone [30]. These authors described the flavonoids of the Bignoniaceae and indicated that flavones of the luteolin and 6-hydroxy-luteolin type are common to the *Catalpa* genus and found in *C. bignonioides*.

More recently, two 5,6-dihydroxy-7,4′-dimethoxyflavone glucosides were described in fruits of *Catalpa bignonioides* Walt [11]. Among flavonoids identified, one corresponds to the 5,6-dihydroxy-7,4′-dimethoxyflavone-6-*O*-sophoroside previously isolated by Oh et al. from *C. bignonioides*. Its fragment ion [M+H]^+^ = 315.0840, corresponding to the ladanein aglycone [34], proves its structure. The other three flavonoids (entries F2–F4) have similar UV spectra and provide the same fragment ion [M+H]^+^ = 303.048, which could allow the aglycone part to be identified as 6-hydroxyluteolin, frequently found in Bignoniaceae and characteristic of catalpa according to Harborne. The putative structures of the three flavonosides would then be 5,6,3′,4′-tetrahydroxy-flavon-7-glucoside [30], 6-hydroxyluteolin-*O*-(-*O*-feruloyl)-glycoside [35], and 6-hydroxyluteolin-*O*-coumaroyl glycoside [36]. To our knowledge, this is the first time that the last two acylated flavone glycosides (F2 and F3 entries, Table 1) have been identified in the *Catalpa* genus.

Other phenolic compounds have also been extracted and identified, including *p*-hydroxybenzoic acid, *p*-coumaric acid, *p*-hydroxybenzoyl-glucopyranoside, martynoside, and a caffeolyl phenylethanoid glycoside compound. These substances have already been described in extracts from species belonging to the *Catalpa* genus [11,29,34].

**Table 1:** Identification of chemical compounds in *Catalpa bignonioides* fruit using ESI-QTOF-MS technique.

Entry	Compounds	Molecular Formula	Molecular Weight	Pseudo-Molecular Ion [M+H]^+^	err [ppm]	mSigma	Score (%)	RT	Fragments Ions [+H]^+^	Samples			Ref.
										EAC	EBC	EDC	
**Flavonoids**													
**F1**	5,6-Dihydroxy-7,4′- dimethoxyflavone- 6-*O*-sophoroside	C_29_H_34_O_16_	638.1920	639.1892	4.4	2.8	100	8.6	315.0840	✓	✓	✓	[11]
**F2**	6-Hydroxyluteolin-*O*- coumaroyl glycoside	C_30_H_26_O_14_	610.13226	611.1374	2.8	9.7	100	9.2	303.0489	✓	✓		[36]
**F3**	6-Hydroxyluteolin- *O*-feruloyl glycoside	C_31_H_28_O_15_	640.14282	641.1494	1.1	8.8	100	8.9	303.0486	✓	✓		[35]
**F4**	5,6,3′,4′-Tetrahydroxy- flavon-7-glucoside	C_21_H_20_O_12_	464.09548	465.1019	1.9	2.7	100	7.7	303.0484	✓	✓		[30]
**Iridoids**													
**I1**	Catalpol	C_15_H_22_O_10_	363.30340	363.1065	2.4	6.5	100	7.9	163.0389	✓			[37]
**I2**	Catalposide	C_22_H_26_O_12_	482.14243	483.1485	2.5	11.5	100	7.8	321.0945 303.0857	✓	✓	✓	[37]
**I3**	Dihydrocatalposide or 6-*O*-*p*-hydroxybenzoyl- 5,7-bisdeoxycynanchoside	C_22_H_28_O_12_	484.15808	485.1685	1.1	6.0	100	7.4	287.0909	✓	✓	✓	[27,28]
**I4**	Verproside	C_22_H_26_O_13_	498.13734	499.1438	4.7	15.8	100	7.1	137.0234 319.0814	✓	✓	✓	[32]
**I5**	6-*O*-(*p*-Hydroxybenzoyl)-descinnamoylglobularimin	C_22_H_28_O_13_	500.1530	501.1583	3.5	10.9	100	6.6	321.0958 483.1483	✓	✓	✓	[27]
**I6**	Verminoside	C_24_H_28_O_13_	524.15299	525.1587	3.1	15.9	100	6.0	163.0389	✓	✓		[32]
**I7**	6-*O-*cis(trans)-*p*-Coumaroyl-5,7-bisdeoxycynanchoside	C_24_H_30_O_12_	510.17373	511.1794	3.2	12.7	100	8.2	-	✓	✓	✓	[28]
**I8**	Minecoside *	C_25_H_30_O_13_	538.16864	539.1745	2.6	18.6	100	8.3	377.1226 177.0543	✓	✓	✓	[11]
**I9**	Picroside III *	C_25_H_30_O_13_	538.16864	539.1743	3.1	8.9	100	8.4	377.1252 177.0545	✓	✓	✓	[11]
**I10**	Specioside	C_24_H_28_O_12_	508.15808	509.1645	1.8	6.8	100	8.6	-	✓	✓	✓	[11]
**Phenolic acid**													
**P1**	*p*-Hydroxybenzoic acid	C_7_H_6_O_3_	138.03169	139.0392	2.3	3.2	100	6.6	121.0399 77.0387 65.0388	✓	✓	✓	[11]
**P2**	*p*-Coumaric acid	C_9_H_8_O_3_	164.04734	165.0546	0.2	13.2	100	1.3	-	✓	✓		[11]
**Phenolic glycosides**													
**P3**	*p*-Hydroxybenzoyl-glycoside	C_13_H_16_O_8_	300.08452	301.0909	3.9	4.5	100	4.5	139.0389	✓	✓		[29]
**P4**	Martynoside	C_31_H_40_O_15_	652.23672	653.2421	2.9	16.0	100	8.9	177.0544	✓	✓	✓	[34]
**P5**	Caffeolyl phenylethanoid glycoside isomer (Verbascoside isomer)	C_29_H_36_O_15_	624.20542	625.2113	2.3	8.5	100	8.1	163.0389	✓	✓	✓	[34]

(*) Interchangeable.

**Table 2:** Retention time (RT) and compounds identified in *Catalpa bignonioides* fruit samples using HPLC-DAD-MS technique.

Peaks	RT	Identification	[M-H]^−^/[M+45]^−^ *	UV λmax (nm)	EAC	EBC	EDC
**1**	3.48	6-*O-*(*p*-Hydroxybenzoyl)-glucoside	299/344	215, 262	✓	✓	✓
**2**	10.83	*p*-Hydroxybenzoic acid	137/183	215, 257	✓	✓	✓
**3**	10.83	6-*O*-(*p*-Hydroxybenzoyl)- descinnamoylglobularimin	499/545	215, 257	✓	✓	✓
**4**	13.90	Dihydrocatalposide or 6-*O*-(*p*-hydroxybenzoyl)- 5,7-bisdeoxycynanchoside	483/529	225, 260	✓	✓	✓
**5**	16.65	Verproside	497/543	224, 265, 300	✓	✓	✓
**6**	20.34	6-Hydroxyluteolin- *O*-feruloyl glycoside	639/685	225, 290, 323	✓	✓	
**7**	22.33	*p*-Coumaric acid	163/209	227, 310	✓	✓	
**8**	24.90	Catalposide	481/527	216, 261	✓	✓	✓
**9**	27.47	5,6,3′,4′-Tetrahydroxy- flavon-7-glucoside	463/509	254, 354	✓	✓	
**10**	28.14	Verbascoside isomer	623/669	224, 300, 330	✓	✓	✓
**11**	29.09	Verminoside	523/569	224, 328	✓	✓	
**12**	33.41	6-*O*-*cis*/*trans*-*p*-Coumaroyl- 5,7- bisdeoxycynanchoside	509/555	224, 302	✓	✓	✓
**13**	35.43	Minecoside **	537/583	224, 328	✓	✓	✓
**14**	37.58	Picroside III **	537/583	224, 328	✓	✓	✓
**15**	38.34	6-Hydroxyluteolin- *O*-coumaroyl glycoside	609/655	227, 258, 355	✓	✓	✓
**16**	39.86	Specioside	507/553	230, 315	✓	✓	✓
**17**	48.40	Martynoside	651/697	224, 323	✓	✓	✓

(*) Formate adduct in ESI^−^; (**) interchangeable.

**Table 3:** Iridoid structures identified in fruits of *Catalpa bignonioides*.

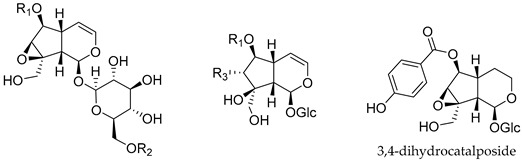			
Compound	R1	R2	R3
Catalpol	H	H	-
Catalposide	4-hydroxybenzoyl	H	-
Verproside	3,4-dihydroxybenzoyl	H	-
Minecoside	3-hydroxy-4-methoxy cinnamoyl	H	-
Specioside	4-hydroxy cinnamoyl	H	-
Verminoside	3,4-dihydroxy cinnamoyl	H	-
Picroside III	H	3-methoxy-4-hydroxy cinnamoyl	-
6-*O*-(*p*-Hydroxybenzoyl)- 5,7-bisdeoxycynanchoside	4-hydroxybenzoyl	-	H
6-*O*-(*p*-Hydroxybenzoyl)- descinnamoylglobularimin	4-hydroxybenzoyl	-	OH
6-*O*-*cis*(*trans*)-*p*-Coumaroyl- 5,7-bisdeoxycynanchoside	4-hydroxy cinnamoyl	-	H

### 3.2. Yields of the Major Iridoids of C. bignonioides Fruit Extracts

The fruits of *Catalpa* contain numerous iridoids. Among the ten identified iridoids, catalpol and catalposide are biosynthesized in significant quantities by *Catalpa bignonioides*.

**Figure 1:**
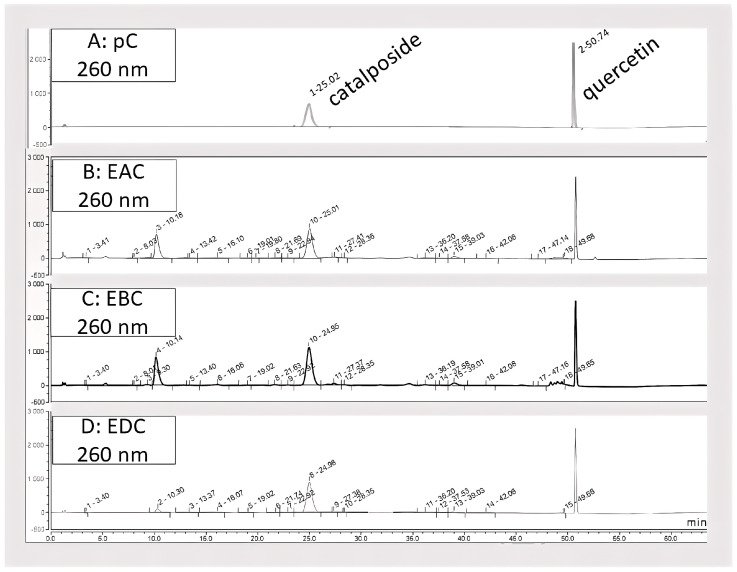
Chromatographic profiles with quercetin (1 g/L) as reference of pure catalposide and of *C. bignonioides* samples using HPLC-DAD-MS technique. (**A**) Chromatogram of pure catalposide (0.8 g/L). (**B**) Chromatogram of EAC (10.2 g/L). (**C**) Chromatogram of EBC (5.0 g/L). (**D**) Chromatogram of EDC (2.4 g/L). After each peak number, retention time is indicated.

Figure 1 shows two significant peaks in the EAC and EBC samples. The peak at 10.8 min corresponds to *p*-hydroxybenzoic acid and hydroxybenzoyl-descinnamoylglobularimine, and the peak at 24.9 min to catalposide. In EDC, the peak at 10.8 min became smaller, indicating that *p*-hydroxybenzoic acid appears to be more soluble in *n*-butanol than catalposide, which precipitates readily in CH_2_Cl_2_. The catalposide dosage showed that the highest amount of catalposide was found in the EDC sample (45%), followed by EBC (28%) and EAC (11%).

The results of dosage analyses carried out on the 2017 batch indicate that fresh *Catalpa* fruits contain 1.37% catalposide. An extract from a batch harvested in August 2023 shows the same chromatographic profiles, but the catalposide content in the fresh fruits is higher (1.7%).

Another abundant iridoid is catalpol, present only in the aqueous extract. As it was not detectable under the recording conditions of our HPLC-DAD-MS experiment, its quantity was estimated after isolating it from the aqueous phase (PA) following extraction with butanol, yielding 1.6% of fresh material.

### 3.3. HPTLC Analyses and Direct Bioautography with DPPH

HPTLC was performed to confirm the presence of acyl-iridoids identified by HPLC analysis in the three samples. In the literature, mobile phases (MP) of different selectivity and polarity have been reported for the separation of iridoid compounds [38].

None of the MPs tested was able to separate the acyl-iridoids identified, due to their structural similarities. However, the MP composed of AcOEt/MeOH/H_2_O (77/15/8 (*v*/*v*/*v*)) with a polarity index of 4.9 was chosen, as it allowed the acyl-iridoids to elute in the middle of the plate, well separated from the migration of catalpol.

The solutions of the three different samples, EAC (100.9 g/L), EBC (12.8 g/L), and EDC (12.7 g/L), were analyzed in the presence of four standard iridoids (catalposide (1.6 g/L), minecoside (1.2 g/L), catalpol (1.1 g/L), picroside III (1.0 g/L)) and two other standards, a phenolic acid (1.3 g/L *p*-hydroxybenzoic acid) and a flavonoid (1.1 g/L rutin).

**Figure 2:**
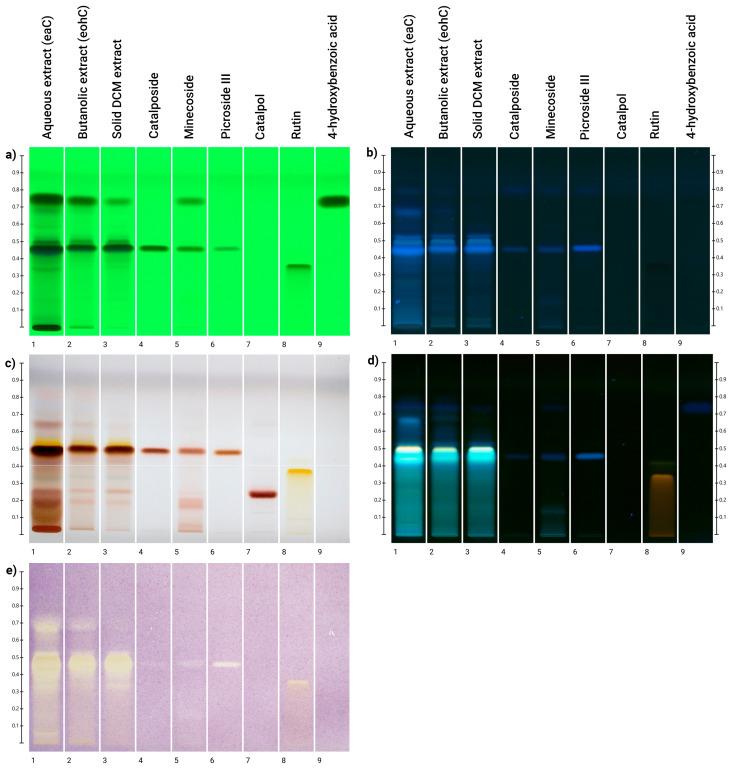
HPTLC fingerprints developed with: (**a**) UV 254 nm; (**b**) UV 366 nm; (**c**) white light vanillin reagent derivatization; (**d**) UV 366 nm NP-PEG derivatization; (**e**) white light DDPH derivatization. (1) EAC 100.9 g/L; (2) EBC 12.8 g/L; (3) EDC 12.7 g/L; (4) catalposide 1.6 g/L; (5) minecoside 1.2 g/L; (6) picroside III 1.0 g/L (7) catalpol 1.1 g/L; (8) rutin 1.1 g/L; (9) *p*-hydroxybenzoic acid 1.3 g/L.

Detection at 254 nm, 366 nm, and after treatment with vanillin-sulfuric acid reagent revealed the presence of the following acyl-iridoids (Rf = 0.48): catalposide (spot 4), minecoside (spot 5), and picroside III (spot 6), in all three extracts (Figure 2a–c).

Unfortunately, it was not possible to discriminate each of them independently. Regarding catalpol (spot 7), revealed with vanillin-sulfuric acid reagent (see Figure 2c), it was mainly present in the aqueous extract (EAC) and did not appear in the *n*-butanol (EBC) and CH_2_Cl_2_ (EDC) samples. A second analysis with NP-PEG reagent (see Figure 2d) revealed the presence of phenol compounds by intense blue fingerprints at the same level as acyl-iridoid compounds. Since the standards were not as luminous as the spots at the same Rf in the three samples, this means that other phenolic compounds are involved. It is also important to note that no yellow/orange color, characteristic of flavonoid compounds, has been observed in the samples.

To determine the antioxidant activity of acyl-iridoids, HPTLC analysis was coupled with a DPPH derivatization reagent [39].

The antioxidant activity of the three samples was confirmed by several yellow bands appearing on the purple background in their tracks (see Figure 2e). Intensive bands of the three samples were located around the catalposide and derivatives area. Standard acyl-iridoids in lower concentrations displayed significant antioxidant activity. These observations lead us to conclude that the anti-free radical activity observed in the extracts is attributable to iridoid derivatives, as well as to compounds with similar Rf values. This technique showed that catalpol, which lacks a phenol group, did not respond to the DPPH test under these conditions. This finding aligns with the work of [40], who reported that catalpol is inactive at a concentration of 200 μM. Flavonoids, martinoside, and phenol compounds found in aqueous extracts are known to participate in the DPPH activity [41].

### 3.4. In Vitro Evaluation of Antioxidant Activity of C. bignonioides

Oxidative stress is defined as the imbalance between the occurrence of reactive oxygen species (ROS) and reactive nitrogen species (RNS) and the ability of endogenous antioxidant systems to counteract their action [42]. Free radical-induced damage in oxidative stress has been confirmed as a contributor to the pathophysiology and pathogenesis of several chronic conditions, including inflammation, cancer, neurodegenerative diseases, and cardiovascular diseases. Antioxidants can minimize oxidative damage. Some phytochemicals, such as polyphenols, carotenoids, and iridoids, have demonstrated great antioxidant potential [43]. Assessing the antioxidant properties of a sample using a single assay is limited, as antioxidants can operate through diverse mechanisms. Since each assay targets specific aspects of antioxidant activity, relying on only one may provide an incomplete value of the sample’s overall antioxidant potential. As a result, in the current study, we investigated the potential antioxidant activity of *C. bignonioides* samples and catalposide by using four in vitro assays, namely the β-carotene bleaching test, FRAP, ABTS, and DPPH assays.

The results are summarized in Table 4. The radical scavenging activity of *C. bignonioides* was assessed by using ABTS and DPPH tests. The *n*-butanol sample (EBC) showed a promising activity in the ABTS test with an IC_50_ value of 0.5 μg/mL, which was approximately ~3 times lower than that of ascorbic acid used as the positive control (IC_50_ of 1.7 μg/mL). In DPPH test, IC_50_ values in the range 15.7–19.7 μg/mL were obtained. These values are better that reported by Xu et al. [44]. In this work, the ethanol extracts of the leaves of *C. ovata*, *C. fargestii*, and *C. bungei* were studied by using DPPH test founding IC_50_ values of 2.35, 2.01, and 1.73 mg/mL. A considerable reducing power was observed in the FRAP tests, where all investigated extracts exhibited a FRAP value higher than those reported for positive control BHT (102.2–102.7 vs. 63.3 μM Fe (II)/g). Conversely, only EAC was able to protect against lipid peroxidation, as assessed by the β-carotene bleaching test, with an IC_50_ value of 7.1 μg/mL after 30 min of incubation.

Herein, the antioxidant activity of catalposide, one of the most abundant iridoids of *C. bignonioides*, was assessed. This iridoid showed antioxidant effects only in the ABTS test with an IC_50_ value of 0.4 μg/mL, a value about 4 times lower than that of the positive control ascorbic acid (1.7 μg/mL). An IC_50_ value of 27.4 μg/mL was found in the β-carotene bleaching test after 30 min of incubation. The anti-radical activity of catalposide was previously investigated by using ABTS and DPPH tests by Lu et al. [45] that reported IC_50_ values of 20.16 μg/mL and 43.83 μg/mL in the DPPH and ABTS tests, respectively. Previously, Moon et al. [46] studied the potential ability of catalposide to protect Neuro 2A cells from oxidative damage through the induction of HO-1 protein expression and HO activity. The treatment of cells with catalposide resulted in a concentration- and time-dependent up-regulation of both HO-1 protein expression and HO activity. Furthermore, catalposide protected the cells from hydrogen peroxide-induced cell death.

The chemical analyses of EAC and EBC extracts, which exhibited the most promising activity in ABTS test and β-carotene bleaching test, respectively, revealed the presence of verminoside, identified for the first time in *C. bignonioides* fruit extracts, catalpol (only in the EAC extract), *p*-coumaric acid, *p*-hydroxybelzoyl-glycoside, 6-hydroxyluteolin-*O*-coumaroyl glycoside, 6-hydroxyluteolin-*O*-feruloyl glycoside, and 5,6,3′,4′-tetrahydroxy-flavon-7-glucoside, not identified in the EDC extract. Among them, verminoside and *p*-coumaric acid have been reported to have antioxidant properties [47,48].

The iridoid catalpol has demonstrated significant antioxidant potential through different mechanisms. It has been shown to reduce lipid peroxidation, protect DNA and proteins from free radical-induced damage, and modulate key cellular pathways such as Nrf2/HO-1 and Keap1/Nrf2/ARE. Additionally, catalpol enhances the body’s endogenous antioxidant defense system by upregulating enzymes including catalase, glutathione peroxidase, and superoxide dismutase [49].

### 3.5. Biological Activity of C. bignonioides Extracts in Cellular Models of Colorectal Cancer

Few manuscripts have described the biological activity of *C. bignonioides,* despite its use in traditional medicine. Muñoz-Mingarro et al., reported in vivo anti-inflammatory activity of crude extracts of leaves and pods [8], while Dvorská et al., described the antioxidant activity of methanolic extracts from different parts of the plant [9]. Recent studies also demonstrated that *C. bignoniodes* fruit extracts improve the proliferation of C2C12 mouse fibroblasts and motor abilities in vivo [10]. A potential use as antidiabetic agents for some constituents of *C. bignonioides* has also been hypothesized [11].

In this study, we evaluated the anti-cancer potential of EAC, EBC, and EDC using in vitro models of colorectal cancer (HT29 and HCT166 cell lines), focusing on inflammation and oxidative stress, key factors that drive the onset and progression of colon cancer [50,51,52,53]. First, in HT29 and HCT116 cell lines, we evaluated the potential cytotoxicity of all extracts using the trypan blue exclusion method and the MTT assay. As shown in Table 5 and Table 6, EAC, EBC, and EDC did not exhibit cytotoxic effects on HT29 cells. A significant decrease in HCT116 cell viability was observed only at the highest concentrations of EBC and EDC, suggesting differential sensitivity among various colorectal cancer cell types.

**Figure 3:**
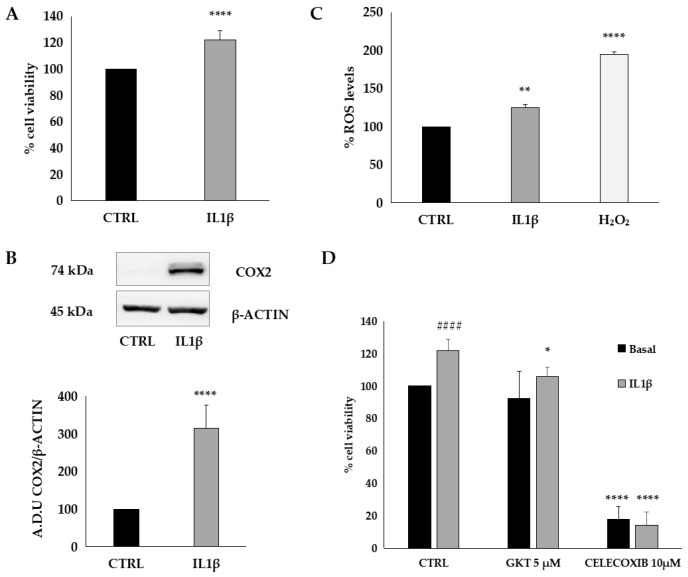
IL1β induces COX2 expression and ROS production. (**A**) Cell viability was measured by MTT assay. Data are reported as the % of cell viability over control and are the means of three experiments run in triplicate. **** *p* < 0.0001 vs. CTRL (**B**) COX2 expression was evaluated by Western blot analysis. The data are representative of three different experiments. **** *p* < 0.0001 vs. CTRL (**C**) ROS measurement was performed by DCFH2-DA assay. Data are expressed as % of ROS levels over control and are representative of three different experiments run in triplicate. ** *p* < 0.01 and **** *p* < 0.0001 vs. CTRL (**D**). The effect of ROS and COX2 on cell vitality was measured after treatment of HT29 cells with GKT137831 or celecoxib #### *p* < 0.001 vs. CTRL, **** *p* < 0.0001 and * *p* < 0.05 vs. IL1β.

To establish a reliable experimental model of inflammation and oxidative stress, HT29 cells were treated with interleukin 1β (IL1β), a key inflammatory mediator (Figure 3). As expected, IL1β promoted cancer cell viability (Figure 3A), increased cyclooxygenase-2 (COX2) expression (Figure 3B), and reactive oxygen species (ROS) production (Figure 3C).

Furthermore, the treatment of HT29 cells with GKT137831 (5 µM), a general NOX inhibitor, or celecoxib (10 µM), a specific COX inhibitor, significantly suppressed the effects of IL1β (Figure 3D), indicating that cell viability is strongly influenced by inflammation and oxidative stress.

**Figure 4:**
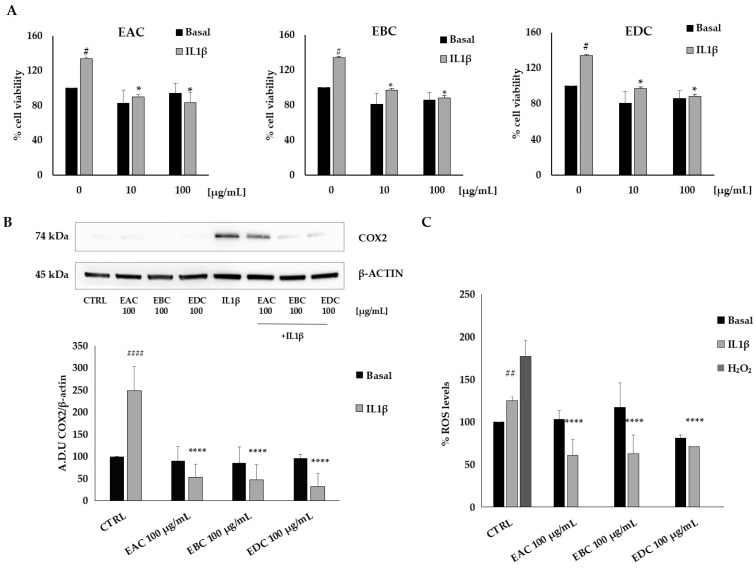
EAC, EBC, and EDC inhibit HT29 cell viability through the inhibition of COX2 expression and ROS production. (**A**) Cell viability was measured by MTT assay. Data are reported as % of cell viability over control and are the means of three experiments run in triplicate. # *p* < 0.05 vs. basal; * *p* < 0.05 vs. IL1β (**B**) Expression levels of COX2 measured by Western blotting. The data are representative of five different experiments. #### *p* < 0.0001 vs. CTRL; **** *p* < 0.0001 vs. IL1β (**C**) ROS measurement was performed by DCFH2-DA assay. Data are expressed as % of ROS levels over control and are representative of three different experiments run in triplicate. ## *p* < 0.01 vs. CTRL; **** *p* < 0.0001 vs. IL1β.

To explore the effects of EAC, EBC, and EDC in inflammation and oxidative stress, we treated HT29 cells with the extracts at concentrations of 10 and 100 μg/mL, 1 h before IL1β exposure. We observed that under these conditions, the effects of the inflammatory stimuli were inhibited (Figure 4).

Specifically, in Figure 4A, we show that the increase in cell viability induced by IL1β was reduced by EAC, EBC, and EAC at both concentrations tested.

Consistently, under similar conditions, all the extracts reduced COX2 expression induced by IL1β (Figure 4B) and ROS production (Figure 4C) with comparable activity. Purified catalposide did not exhibit significant activity under the experimental conditions used. These data suggest that *C. bignonioides* extracts inhibit inflammation and oxidative stress in colorectal cancer cells, thereby reducing cancer cell viability.

**Figure 5:**
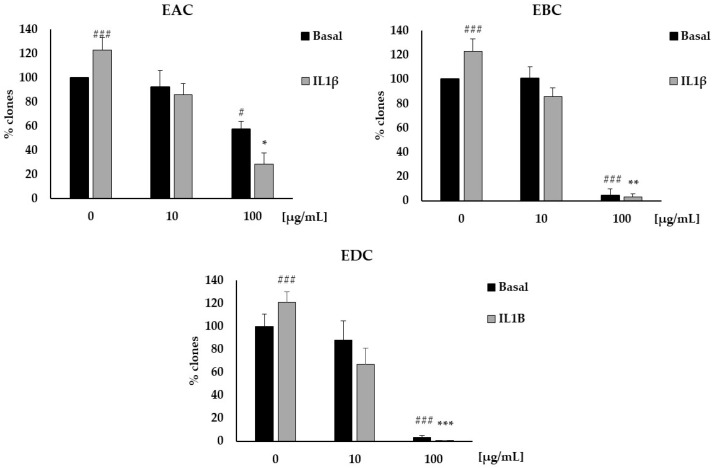
EAC, EBC, and EDC inhibit cell clonogenicity. Cell clonogenicity was reported as a percentage of colonies of HT29 cells in response to IL1β 10 ng/mL and EAC, EBC, or EDC (10–100 μg/mL). Data are expressed as % over basal control and are representative of three independent experiments run in triplicate. # *p* < 0.05 and ### *p* < 0.001 vs. basal; * *p* < 0.05, ** *p* < 0.01 and *** *p* < 0.001 vs. IL1β.

Finally, to further explore the potential anti-cancer activity of EAC, EBC, and EDC, we performed a clonogenic assay to evaluate the effects of the extracts on the clonogenic growth of cancer cells. IL1β induced a significant increase in the number of clones of HT29 cells, which was strongly inhibited by all the extracts (Figure 5), but not by purified catalposide. Importantly, we also observed a potent effect of EAC, EBC, and EDC under basal conditions at 100 μg/mL, suggesting that *C. bignonioides* fruit extracts exert anti-cancer activity even in the absence of specific stimuli.

Under our experimental conditions, all three *C. bignonioides* extracts studied have been shown to reduce the viability of colon cancer cells by inhibiting the COX-2 mediated inflammatory process and intracellular ROS levels, demonstrating anti-inflammatory and antioxidant activity, and confirming in a cellular model the data obtained by chemical assays. The *C. bignonioides* extracts also inhibited cancer cell clonogenicity both under basal conditions and in IL1β-stimulated cells. In all the experiments conducted, no significant differences were observed among the three extracts, despite their different compositions of active compounds. EAC was found to be the richest in metabolites and contains catalpol, which has been shown to exhibit multiple biological effects, including anti-cancer properties. The common denominator of the various activities of catalpol appears to be its anti-inflammatory and antioxidant activity [54,55,56,57]. However, catalpol has not been detected in EBC and EDC extracts.

Among the ten iridoids detected in the EAC extract, nine were acyl-iridoids, and the most abundant was catalposide, whose concentration increased during the purification process, reaching 45% in EDC. Different studies report the anti-inflammatory, antioxidant, and anti-cancer activity of catalposide [44,58,59,60,61,62]. However, in our experimental model and at the concentrations used, pure catalposide did not show activity in the biological assays. Even in chemical assays for determining antioxidant activity, except the ABTS assay, pure catalposide had shown low antioxidant activity compared to *Catalpa* extracts.

In addition to catalposide, two other acyl-iridoids, minecoside and picroside III, were also detected in all the extracts. Interestingly, minecoside has been shown to possess anticancer and anti-metastatic properties in breast and colon cancer cells [63,64], while picroside III has been found to inhibit intestinal inflammation by suppressing the PI3K-AKT pathway [65] and to protect intestinal barrier integrity through AMPK activation [66].

We identified for the first time verproside and verminoside in the *C. bignonioides* fruit extracts. Both verproside and verminoside have been reported to have anti-inflammatory properties in in vitro experiments and in in vivo models [67,68,69].

Studies conducted using both isolated compounds and plant extracts (*Veronica officinalis, V. ciliata, V. incana, Pseudolysimachion rotundum*), whose main components were verproside and verminoside, have demonstrated that anti-inflammatory effects are mediated by the inhibition of proinflammatory mediators via the NF-kB signaling pathway [61,69,70,71,72]. Further in vitro and in vivo investigations have demonstrated that active fractions from *Veronica ciliata,* mainly containing verproside and catalposide, have significant anti-hepatocarcinoma activity and antioxidant properties. The protective mechanism involves the p62-Keap1 Nrf2 signaling pathway. [44,60]. Finally, a methanolic extract from the leaves of *Pseudolysimachion rotundum* and verminoside isolated from the plant demonstrated anticancer effects both in vitro and in vivo [73].

## 4. Conclusions

We conducted a thorough characterization of the phytochemical profile of *C. bignonioides* extracts. Aqueous extract of *C. bignonioides* fruits showed a few flavonoids and a rich variety of acyl-iridoids with hydroxybenzoyl or hydroxycinnamoyl groups. Catalpol and catalposide were present in high concentrations. Interestingly, we found that EAC and EBC extracts contain verproside and verminoside, which have not previously been identified in *Catalpa bignonioides* extracts, as well as two flavonoid glycosides that have not been reported before in the *Catalpa* genus. Of the phenolic acids and glycosides, *p*-hydroxybenzoic acid was the most abundant. The aqueous fruit extraction process, followed by a *n*-butanol extraction of the EAC extract and treatment of the latter with dichloromethane, yielded an acyl-iridoid-enriched EDC precipitate with abundant catalposide.

HPTLC analysis coupled with a DPPH derivatization reagent showed the involvement of iridoids in the antioxidant effect. In addition, the three extracts exhibited distinct antioxidant activities when evaluated through in vitro assays, a finding attributable to their different chemical composition.

In vitro studies on colon cancer cell models revealed that *Catalpa* extracts possess potential anticancer properties, likely mediated by their anti-inflammatory and antioxidant effects. Across all conducted experiments, no statistically significant differences were observed among the three extracts, despite their distinct profiles of bioactive constituents.

These data strongly suggest that the biological activities demonstrated in this study cannot be ascribed to a single compound but rather result from the synergistic contribution of all the bioactive constituents present. The identified iridoids—and potentially their metabolites generated within the cellular model—undoubtedly play a pivotal role. Nonetheless, the contribution of other bioactive molecules, such as flavonoids and phenolic derivatives, should not be underestimated.

## Figures and Tables

**Table 4:** Antioxidant activity of *C. bignonioides* fruit extracts and catalposide.

Sample	DPPH Test IC_50_ (μg/mL)	ABTS Test IC_50_ (μg/mL)	β-Carotene Bleaching Test ^#^ IC_50_ (μg/mL)	FRAP Test * μM Fe(II)/g
EAC	19.7 ± 1.9 **	1.2 ± 0.3 ^ns^	7.1 ± 0.8 ***	102.7 ± 7.8 ***
EBC	16.9 ± 1.6 *	0.5 ± 0.04 ***	NA	102.2 ± 7.5 ***
EDC	15.7 ± 1.7 *	1.1 ± 0.1 *	NA	102.6 ± 7.7 ***
Catalposide	60.5 ± 5.3 ***	0.4 ± 0.03 ***	27.4 ± 4.6 ***	14.8 ± 1.8 ***
Positive control				
Ascorbic acid	5.3 ± 0.6	1.7 ± 0.5		
BHT				63.3 ± 3.9
Propyl gallate			1.2 ± 0.4	

Data are expressed as mean ± standard deviation (*n* = 3). NA: not active at the maximum concentration tested. * tested at the concentration of 2.5 mg/L. # t = 30 min of incubation. One-way ANOVA *** *p* < 0.0001 followed by a multicomparison Dunnett’s test: *** *p* < 0.0001; ** *p* < 0.001; * *p* < 0.01 ***; ns: not significant.

**Table 5:** EAC, EBC, and EDC do not induce cell death. Cell toxicity of EAC, EBC, and EDC at different concentrations was evaluated by trypan blue assay. The results are expressed as the percentage of dead cells relative to the total number of cells and are representative of three different experiments run in triplicate. CTRL: untreated cells.

HT29					HCT116			
Concentration (µg/mL)	CTRL	EAC	EBC	EDC	CTRL	EAC	EBC	EDC
**0.01**	7.1% ± 1.7	3.6% ± 0.8	1.6% ± 0.5	6.9% ± 4.6	7.6% ± 1.7	12% ± 2.9	16.3% ± 1.8	5.1% ± 2.6
**0.1**		18.9% ± 1.2	1.8% ± 0.4	12.7% ± 2.3		17% ± 2.1	10.6% ± 3.7	11.4% ± 5.3
**1**		4.8% ± 0.5	5.3%± 0.5	8.3% ± 3.0		11.5% ± 2.4	17.6% ± 7.6	9.9% ± 6.0
**10**		15.9% ± 1.3	4.8% ± 0.8	12.8% ± 3.4		7.6% ± 2.1	6.7% ± 1.67	14.3% ± 1.4
**100**		5.9% ± 0.8	2.6% ± 0.8	15.0% ± 4.5		7% ± 1.3	16.4% ± 5.9	13.0% ± 5.5

**Table 6:** EAC, EBC, and EDC do not reduce cell viability in basal conditions. Cell viability was measured by MTT assay. The results are expressed as the percentage of cell viability relative to the control and are representative of three different experiments run in triplicate. CTRL: untreated cells.

		HT29			HCT116		
Concentration (µg/mL)	CTRL	EAC	EBC	EDC	EAC	EBC	EDC
**0.01**	100%	98.5 ± 11.7	112.6 ± 10.8	111.1 ± 4.5	99.5 ± 11.8	106.0 ± 7.2	101.0 ± 3.2
**0.1**		88.7 ± 12.6	101.6 ± 8.7	99.4 ± 4.4	80.3 ± 3.0	80.5 ± 9.0	82.2 ± 4.1
**1**		100.3 ± 2.5	88.8 ± 1.7	105.5 ± 2.9	100.2 ± 11.9	104.9 ± 6.3	100.3 ± 10.4
**10**		94.7 ± 14.2	94.3 ± 15.2	101.1 ± 9.0	87.5 ± 1.8	82.6 ± 1.5	85.4 ± 1.9
**100**		90.7 ± 7.5	84.3 ± 13.4	103.3 ± 2.5	94.0 ± 11.8	56.5 ± 12.2 *	66.5 ± 10.2 *

* *p* < 0.05 vs. CTRL.

## Data Availability

All data generated or analyzed during this study are available from the corresponding author upon reasonable request.

## Supplementary Materials

The following supporting information can be downloaded at https://www.mdpi.com/article/10.3390/antiox14091116/s1 Chemical section, NMR data (Figure S1: ^1^H and ^13^C NMR spectra of catalpol [74,75]; Figure S2: ^1^H and ^13^C NMR spectra of catalposide [76]. Figure S3: ^1^H and ^13^C NMR spectra of specioside [77]. Figure S4: ^1^H and ^13^C NMR spectra of minecoside) [77].
