# Supplementary material for: A Study of the Fruits of Catalpa bignonioides Walt.: Evaluation of the Antioxidant, Anti-Inflammatory, and Anti-Cancer Activities in Colorectal Adenocarcinoma Cells in Relation to Phytochemical Profile
Source: Antioxidants (Basel). 2025 Sep 14;14(9):1116. doi: 10.3390/antiox14091116 (PMC12466890; doi:10.3390/antiox14091116)
Supplement: Supplementary file 1 [file antioxidants-14-01116-s001.zip › antioxidants-3696274-supplementary.pdf]

A study of the Fruits of *Catalpa bignonioides* Walt.: Evaluation of the Antioxidant, Anti-inflammatory, and Anti-cancer Activities in Colorectal Adenocarcinoma Cells in relation to phytochemical profile

Clizia Bernardi [1], Thomas Gaslonde [2,†], Federica Finetti [1,†], Salim Benmaouche [2], Giulia Macrì [1,2], Annabelle Dugay [2], Claire Cuyamendous [2], Chouaha Bouzidi [2], Monica Rosa Loizzo [3], Philippe Belmont [2], Rosa Tundis [3], Lorenza Trabalzini [1] and Brigitte Deguin [2,*]

[1] Department of Biotechnology, Chemistry and Pharmacy, University of Siena, 53100 Siena, Italy; clizia.bernardi2@unisi.it (C.B.); federica.finetti@unisi.it (F.F.); giulia.macri@vismederi.com (G.M.); lorenza.trabalzini@unisi.it (L.T.)
[2] Faculté de Pharmacie de Paris, Université Paris Cité, U.M.R. CiTCoM (n°8038—CNRS/Université Paris Cité), F-75006 Paris, France; thomas.gaslonde@u-paris.fr (T.G.); salim.benmaouche@u-paris.fr (S.B.); annabelle.dugay@u-paris.fr (A.D.); claire.cuyamendous@u-paris.fr (C.C.); chouaha.bouzidi@u-paris.fr (C.B.); philippe.belmont@u-paris.fr (P.B.)
[3] Department of Pharmacy, Health and Nutritional Sciences, University of Calabria, 87036 Rende, Italy; monica_rosa.loizzo@unical.it (M.R.L.); rosa.tundis@unical.it (R.T.)
* Correspondence: brigitte.deguin@u-paris.fr
† These authors contributed equally to this work.

1. Chemical section, NMR data

Figure S1 : ^{1}H and ^{13}C NMR spectra of Catalpol
Figure S2 : ^{1}H and ^{13}C NMR spectra of Catalposide
Figure S3 : ^{1}H and ^{13}C NMR spectra of Specioside
Figure S4 : ^{1}H and ^{13}C NMR spectra of Minecoside

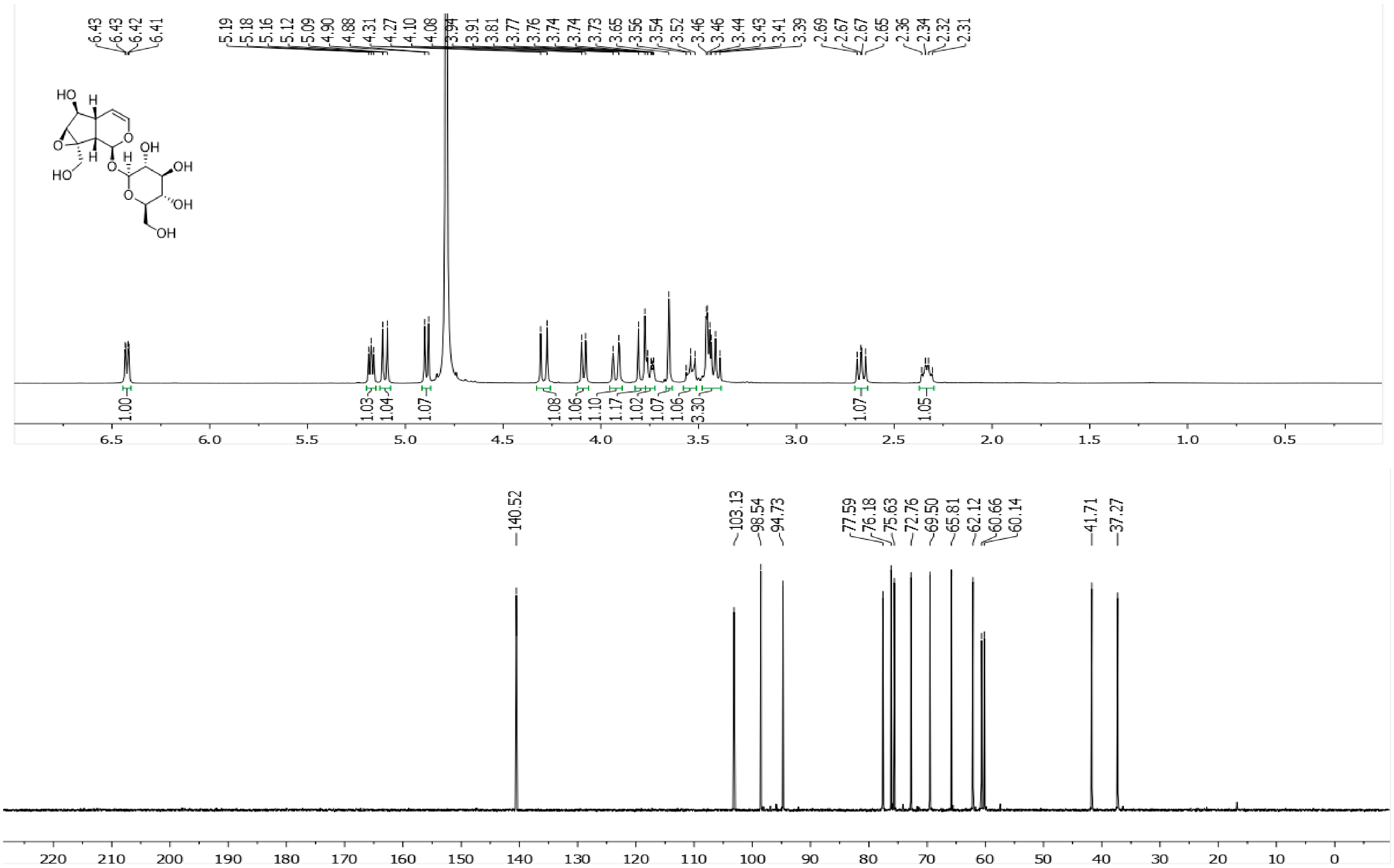

Figure S1: ^{1}H and ^{13}C NMR spectra of catalpol (In agreement with literature:[74,75])

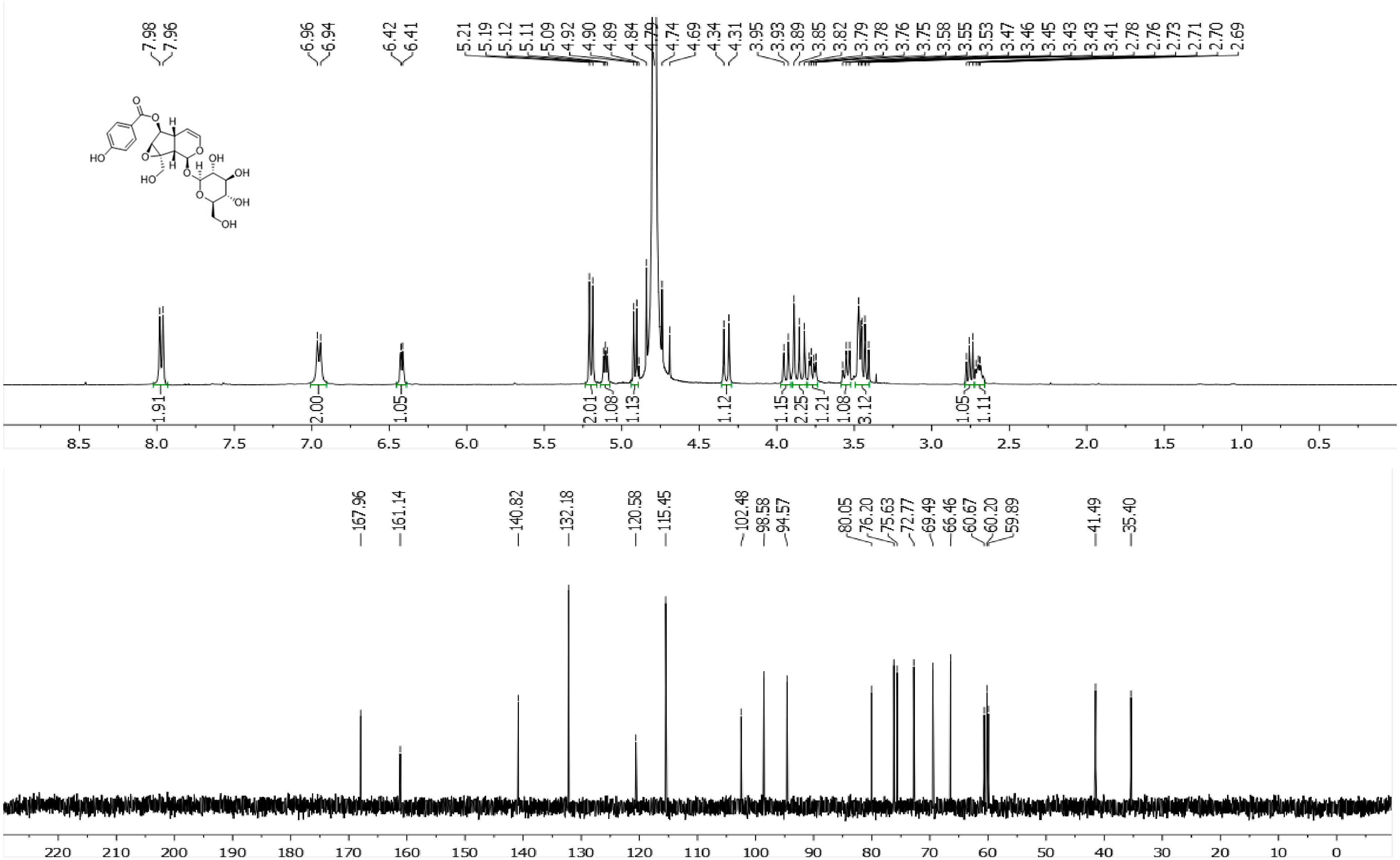

Figure S2: ^{1}H and ^{13}C NMR spectra of catalposide (In agreement with literature:[76])

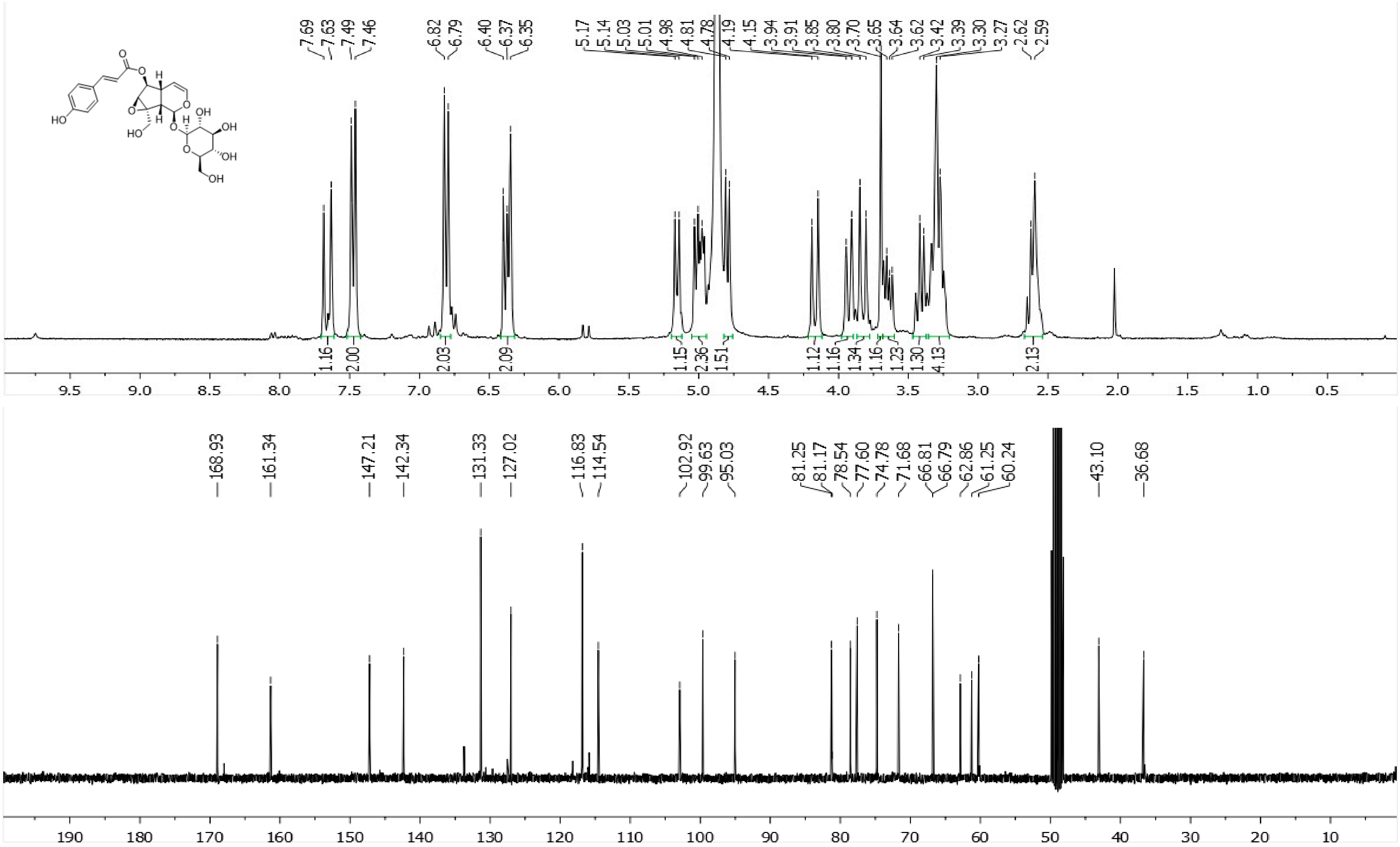

Figure S3: ^{1}H and ^{13}C NMR spectra of specioside (In agreement with literature: [77])

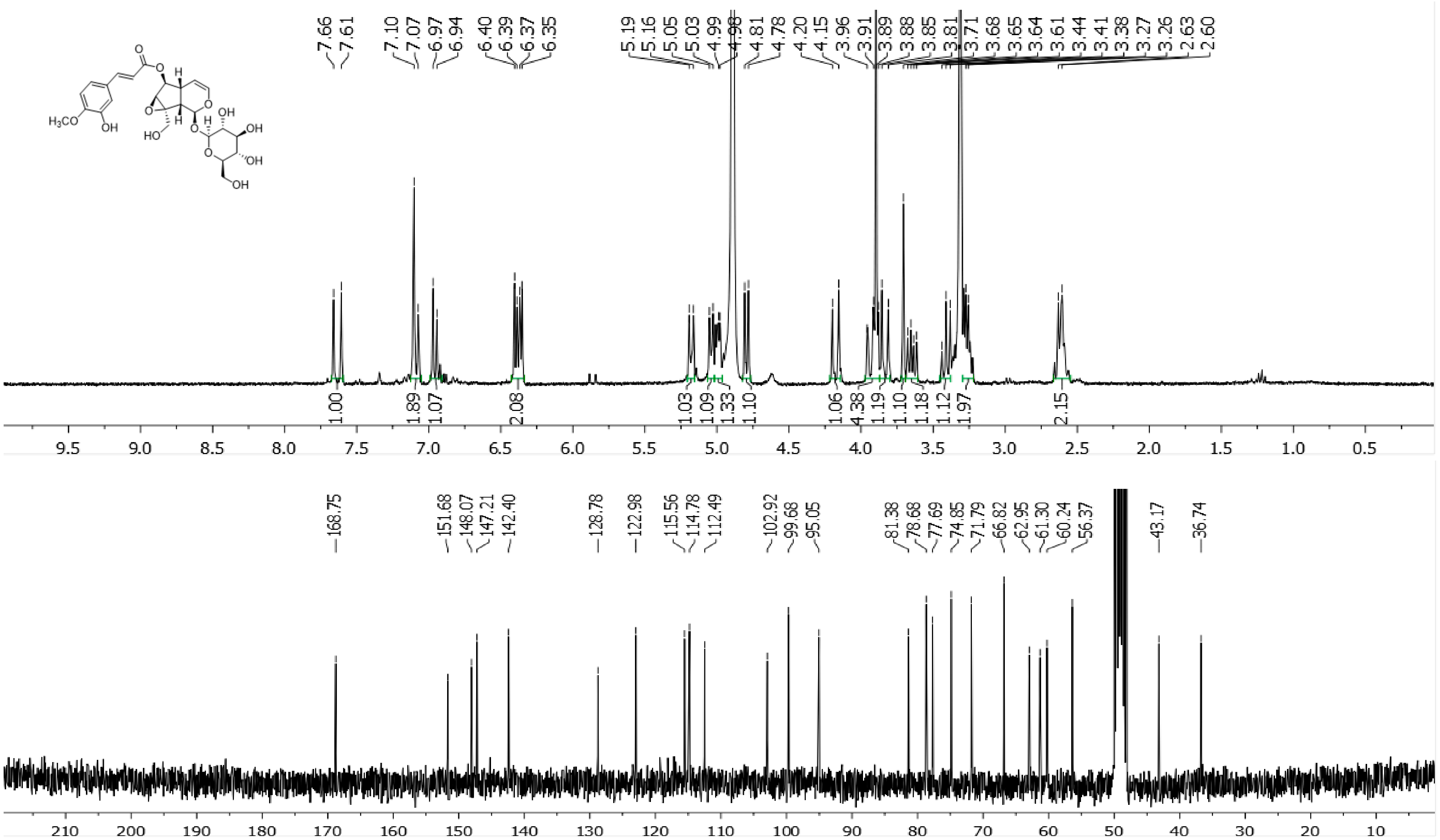

Figure S4: ^{1}H and ^{13}C NMR spectra of minecoside (In agreement with literature:[77])

74. Frezza, C.; De Vita, D.; Venditti, A.; Baldani, C.; Giampaoli, O.; Sciubba, F.; Bosco, C. D.; Franceschin, M.; Beccaccioli, M.; Reverberi, M.; Percaccio, E.; Di Sotto, A.; Foddai, S. Phytochemical analysis and biological activities of the aerial parts of *Odontites Vulgaris* Moench. *Fitoterapia* **2024**, *175*, 105936. https://doi.org/10.1016/j.fitote.2024.105936.
75. Ramírez-Cisneros, M. Á.; Rios, M. Y.; Aguilar-Guadarrama, A. B.; Rao, P. P. N.; Aburto-Amar, R.; Rodríguez-López, V. In Vitro COX-1 and COX-2 Enzyme inhibitory activities of iridoids from *Penstemon Barbatus*, *Castilleja Tenuiflora*, *Cresentia Alata* and *Vitex Mollis*. *Bioorg. Med. Chem. Lett.* **2015,** *25*, 4505–4508. https://doi.org/10.1016/j.bmcl.2015.08.075.
76. Saracoglu, I.; Suleimanov, T.; Pashaeva, N.; Dogan, Z.; Inoue, M.; Nakashima, K. Iridoids from *Veronica Crista-Galli* from the Flora of Azerbaijan. *Chem. Nat. Compd*. **2020**, *56*, 751–753. https://doi.org/10.1007/s10600-020-03139-3.
77. Phuong Thao, T. T.; Bui, T. Q.; Quy, P. T.; Bao, N. C.; Van Loc, T.; Van Chien, T.; Chi, N. L.; Van Tuan, N.; Van Sung, T.; Ai Nhung, N. T. Isolation, semi-synthesis, docking-based prediction, and bioassay-based activity of *Dolichandrone spathacea* iridoids: New catalpol derivatives as glucosidase inhibitors. *RSC Adv*. **2021**, *11*, 11959–11975. https://doi.org/10.1039/D1RA00441G.
